# Public health in genetic spaces: a statistical framework to optimize cluster-based outbreak detection

**DOI:** 10.1093/ve/veaa011

**Published:** 2020-03-13

**Authors:** Connor Chato, Marcia L Kalish, Art F Y Poon

**Affiliations:** v1 Department of Pathology and Laboratory Medicine, Western University, Dental Sciences Building DSB4044, London N6A 5C1, Canada; v2 Division of Infectious Diseases, Department of Medicine, Vanderbilt University School of Medicine, 1161 21st Ave S, Nashville, TN 37232, USA; v3 Department of Applied Mathematics, Western University, Middlesex College MC255, London N6A 5B7, Canada; v4 Department of Microbiology and Immunology, Western University, Dental Science Building DSB3014, London N6A 5C1, Canada

**Keywords:** molecular epidemiology, genetic clustering, modifiable areal unit problem, virus evolution, HIV prevention

## Abstract

Genetic clustering is a popular method for characterizing variation in transmission rates for rapidly evolving viruses, and could potentially be used to detect outbreaks in ‘near real time’. However, the statistical properties of clustering are poorly understood in this context, and there are no objective guidelines for setting clustering criteria. Here, we develop a new statistical framework to optimize a genetic clustering method based on the ability to forecast new cases. We analysed the pairwise Tamura-Nei (TN93) genetic distances for anonymized HIV-1 subtype B *pol* sequences from Seattle (*n *=* *1,653) and Middle Tennessee, USA (*n *=* *2,779), and northern Alberta, Canada (*n *=* *809). Under varying TN93 thresholds, we fit two models to the distributions of new cases relative to clusters of known cases: 1, a null model that assumes cluster growth is strictly proportional to cluster size, i.e. no variation in transmission rates among individuals; and 2, a weighted model that incorporates individual-level covariates, such as recency of diagnosis. The optimal threshold maximizes the difference in information loss between models, where covariates are used most effectively. Optimal TN93 thresholds varied substantially between data sets, e.g. 0.0104 in Alberta and 0.016 in Seattle and Tennessee, such that the optimum for one population would potentially misdirect prevention efforts in another. For a given population, the range of thresholds where the weighted model conferred greater predictive accuracy tended to be narrow (±0.005 units), and the optimal threshold tended to be stable over time. Our framework also indicated that variation in the recency of HIV diagnosis among clusters was significantly more predictive of new cases than sample collection dates (ΔAIC > 50). These results suggest that one cannot rely on historical precedence or convention to configure genetic clustering methods for public health applications, especially when translating methods between settings of low-level and generalized epidemics. Our framework not only enables investigators to calibrate a clustering method to a specific public health setting, but also provides a variable selection procedure to evaluate different predictive models of cluster growth.

## 1. Background

Spatiotemporal clustering is a fundamental public health methodology for the detection of infectious disease outbreaks (Robertson et al. 2010). The colocalization of cases in space and time can reveal the existence of a common source, and cases within a cluster tend to be related by recent transmission events. For example, an automated time–space clustering method (Kulldorff et al. 2005) was demonstrated to retrospectively detect outbreaks of nosocomial bacterial infection in a US-based hospital, including the outbreaks that were detected contemporaneously by the hospital’s pre-existing infection control programme (Huang et al. 2010). At a broader spatial scale, the same clustering method was recently used to identify outbreaks of severe acute respiratory infections over a five-year period, using case data from a network of hospitals in Uganda (Cummings et al. 2019). Early detection of a cluster represents a potential opportunity for a targeted public health response to prevent additional cases. Time–space clustering may be less effective, however, for pathogens that can establish a chronic infection with a long asymptomatic period (e.g. *Mycobacterium tuberculosis*, hepatitis C virus, or human immunodeficiency virus type 1; HIV-1) where the transmission event may have occurred months or years before diagnosis. Furthermore, pathogens with a relatively low per-act transmission rate present difficulties for time–space clustering because a single exposure in a specific location is unlikely to result in transmission. Under these circumstances, the spread of an epidemic is more likely to be shaped by a social network of repeated contacts between individuals, rather than shared venues.

For many infectious diseases, the molecular evolution of the pathogen is sufficiently rapid that genetic differences can accumulate between related infections on a similar time scale as disease transmission. Consequently, it can be effective to cluster cases in a high-dimensional *genetic space* in addition to clustering in physical space and time. In these studies, a case of infection is represented by a pathogen-derived molecular sequence that maps to some point in genetic space, and it may be associated with subject-level metadata such as the diagnosis date or treatment history. Clustering infections by their evolutionary relatedness is a popular method to identify and characterize subgroups with potentially elevated transmission rates. For example, pairs of sequences can be clustered if the number of genetic differences between them falls below some threshold. The resulting clusters are often visualized as a network or undirected graph, where each node (vertex) represents an individual case of infection, and each edge connecting vertices indicate that the sequences of the corresponding cases are within a threshold genetic distance of each other. Sampling a group of cases that are nearly genetically identical implies that they are related through an unknown number of recent and rapid transmission events. A substantial number of genetic clustering studies have focused on the molecular epidemiology of HIV-1 (Poon 2016; [Bibr veaa011-B37][Bibr veaa011-B34]; Billock et al. 2019). Under current global treatment and prevention guidelines (Levi et al. 2016), greater proportions of HIV cases are being diagnosed, and new diagnoses are more frequently screened for drug resistance by genetic sequencing prior to initiating antiretroviral treatment. As a result, public health organizations are beginning to use genetic clustering methods in ‘near real-time’ to identify ongoing HIV-1 outbreaks (Poon et al. 2016; Gonsalves and Crawford 2018), to reconstruct the risk factors and aetiology (Dennis et al. 2012; Poon et al. 2015), and to prioritize groups for prevention initiatives such as access to pre-exposure prophylaxis (PrEP; Volz et al. 2018).

A fundamental challenge in the use of genetic clustering to identify potential outbreaks is that these methods usually require the specification of one or more clustering criteria (Poon 2016; Hassan *et al.* 2017). Many HIV-1 studies that employ pairwise clustering use similar genetic distance thresholds, where a genetic distance is a measure that adjusts for the possibility of multiple substitutions at the same nucleotide. For instance, a recent review (Hassan *et al.* 2017) of 105 articles defining HIV-1 clusters found that 1.5 per cent was the most commonly used threshold among 52 studies that employed a genetic distance. In contrast, the United States Centers for Disease Control and Prevention (US-CDC) currently recommends a stricter pairwise distance threshold of 0.5 per cent (National Center for HIV/AIDS, Viral Hepatitis, STD, and TB Prevention 2018) for the purpose of identifying clusters with the most recent and rapid growth. In some cases the selected threshold is informed by the expected divergence between HIV-1 sequences sampled longitudinally from the same patient (Poon *et al.* 2015; Wertheim *et al.* 2017)—however, this empirical distribution can vary substantially among subjects (Ratmann *et al.* 2016) and may be influenced by the extent of clinical follow-up. In other cases, the threshold is based on the expected number of substitutions between two epidemiologically unrelated individuals in the same sample space (∼5% for the USA; Aldous *et al.* 2012; Oster *et al.* 2018), which makes the optimal threshold sensitive to regional variation in HIV prevalence and population density. On the other hand, population studies in Botswana (Novitsky *et al.* 2014), South Africa (De Oliveira *et al.* 2017), and South America (Junqueira *et al.* 2016) have used substantially higher distance thresholds (10%, 4.5%, and 4.5%, respectively) that imply the optimal thresholds may vary substantially among settings and HIV-1 subtypes (Hemelaar 2013). Furthermore, simulation-based studies (Novitsky *et al.* 2014; Poon 2016; Dasgupta *et al.* 2019) have demonstrated that clustering is highly sensitive to the sampled proportion of the infected population. Given the known differences in the empirical distributions of HIV-1 genetic distances among populations, as well as the significant global disparities in prevalence and access to testing and treatment, it is urgently necessary to establish an objective, quantitative framework for optimizing a clustering method to the target population.

Here we propose that the most useful approach to select clustering criteria is to base this decision on our ability to predict where the next cases will occur. A high, permissive clustering threshold tends to result in a single cluster that comprises the majority of known cases. The next cases are proportionately more likely to connect to this cluster simply because it is large, but its size will also average out the individual- and group-level attributes that are informative for predicting the next cases. Put another way, a single large cluster is not likely to confer a public health benefit because it is akin to prioritizing the entire population. Conversely, setting a low, strict clustering threshold results in a large number of small clusters. This increases the variation of attributes among clusters, resolving greater information. As cluster sizes continue to decline with progressively lower thresholds, however, the variation in attributes among clusters is less associated with the emergence of new cases—in other words, the distribution of new cases among clusters becomes increasingly random. This trade-off is analogous to the modifiable areal unit problem (MAUP), a concept in spatial statistics first fully conceptualized by Openshaw and Taylor (1979). Areal units are derived from a partition of a geographic range by drawing boundaries that separate households or neighbourhoods. The MAUP formally recognizes the inconsistency of statistical associations with changing boundaries. For example, aggregating units into larger spatial units, such as cities or countries, can prevent an investigator from detecting a strong association between water quality and gastrointestinal illness (Swift, Liu, and Uber 2008).

To address the MAUP in the context of genetic clustering and public health, we develop an information criterion-based framework inspired by work from Nakaya (2000). The objective of our framework is to identify the clustering criteria that maximizes the information content of the resulting clusters for forecasting where the next cases will occur. We evaluate our approach on anonymized HIV-1 sequence data from three populations, and demonstrate how this framework can also be used to select between predictive models of cluster growth that utilize different cluster attributes. Furthermore, we examine the problems associated with the selection of clustering criteria or the application of criteria from one population to another, and evaluate the stability of information-optimized criteria for a given population over time.

## 2. Methods

### 2.1 Data collection and processing

**Figure 1.**
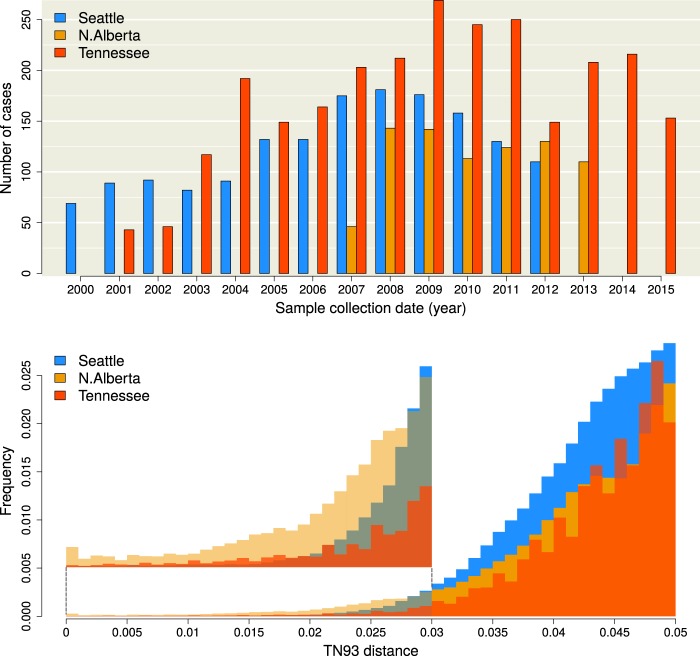
(top) Distribution of sample collection years for the Seattle (blue), northern Alberta (orange), and Middle Tennessee (red) data sets. Absent bars indicate that no sampling was carried out in the respective years, and does not reflect an absence of cases. (bottom) Histograms of Tamura-Nei (TN93) genetic distances among pairwise comparisons of HIV-1 sequences. The height of each bin has been rescaled to reflect the total number of pairwise comparisons, for which the majority were censored from the data. An expanded section of the barplots in the range (0, 0.03) is provided as a figure inset to clarify differences among the distributions.

From the public GenBank database (https://www.ncbi.nlm.nih.gov/genbank, last accessed 2 April 2019), we obtained *n *=* *809 anonymized HIV-1 *pol* sequences sampled from northern Alberta, Canada, between 2007 and 2013 (Vrancken *et al.* 2017), and *n *=* *1,653 sequences collected from Seattle, USA, between 2000 and 2013 (Wolf *et al.* 2017). In addition, *n *=* *2,779 HIV-1 *pol* sequences predominantly from the middle Tennessee region of the USA were collected by the Vanderbilt Comprehensive Care Clinic in Nashville between 2001 and 2015. These data were linked to patient records to extract limited data (e.g. year of HIV-1 diagnosis) and anonymized before being made available for this study; further details are available in Dennis *et al.* (2018). All sequence data were annotated with years of sample collection. We used the pre-existing HIV subtype annotations from the sequence records to filter each data set for non-B subtypes, and excluded repeated samples from the same individual. Next, we filtered each data set to remove any sequences with a proportion of ambiguous nucleotides above 5 per cent, which affected 1 sequence from each of the northern Alberta and Seattle data sets, and 163 sequences from Tennessee. Given the relatively small number of sequences collected in part of 2013 for the Seattle data set (*n *=* *35, Fig. 1), we excluded this year to maintain a consistent sampling rate. We retrieved the sample collection dates for Seattle and North Alberta by querying GenBank with the respective accession numbers and extracting this information from the XML stream returned from the server using the BioPython module (Cock *et al.* 2009) in Python. Next, we used an open-source implementation of the Tamura and Nei (1993) genetic distance in C++ (TN93 version 1.0.6, https://github.com/veg/tn93, last accessed 5 September 2018) for each data set to compute these distances between all pairs of sequences. All other options for the TN93 analyses were set to the default values.

### 2.2 Defining clusters

Using a custom script in the R programming language, we generated an undirected graph G=(V,E) from the TN93 output of each data set, where the set of vertices V represents individual cases (assuming one sequence per case) connected by edges in the set E. Every edge between vertices *v* and *u*, denoted e(v,u)∈E, was weighted with the TN93 distance between the respective sequences, which we denote by the edge attribute *d*(*v*, *u*). In addition, each vertex v∈V carries a temporal attribute *t*(*v*), which may represent the year of diagnosis or sample collection. Note that we are not limited to analysing dates at the level of years and can utilize more precise time intervals, e.g. quarters or months, given the availability of these data. For a given clustering threshold $d_{\text{max}}$, we obtained a spanning subgraph $G_{d}=(V,E_{d})$ from G that results from filtering the complete list of n(n−1)/2 edge weights, such that $E_{d}=\{e(v,u)\in E:d(v,u)\leq d_{\text{max}}\}$. The subset of sequences with the most recent collection time-point was specified as $V=\{v\in V:t(v)=t_{\text{max}}\}$, such that the total number of new cases is |V|. In other words, sampling time cuts V into disjoint vertex sets *V* and *V*^c^, where *V*^c^ is the complement of *V* ($V^{c}\cup V=V$) and contains all known cases over time $t<t_{\max}$. Later it will be useful to refer to the subset of edges in *E*_d_ that connect a vertex in *V*^c^ and a vertex in *V*, which we denote as $E=\{e(v,u):e\in E_{d},v\in V^{c},u\in V\}$. The set of edges in *E* can also be interpreted as edges in a bipartite subgraph comprising parts *V*^c^ and *V*.

A clustering method defines a partition on the known cases *V*^c^ into a set of clusters $\left\{C_{1},C_{2},\ldots ,C_{n}\right\}$ such that $C_{i}\cap C_{j}=\emptyset$ for all i≠j and 1≤i,j≤n; and such that the union of all clusters recovers the entire set: $\overset{n}{\underset{i=1}{\cup }}C_{i}=V^{c}$. Note that this definition does not strictly require the existence of edges, which we use to represent genetic similarity, but can be adapted to any method that defines a partition on the database of known cases. For our analysis, clusters were defined as the connected components of *V*^c^, meaning any pair of vertices within the same cluster ($v,u\in C_{i}$) are connected by at least one path (a sequence of edges), any pair of vertices in different clusters are not connected by any path, and single cases can count as their own cluster of size one.

### 2.3 Modelling growth

We define total cluster growth *R* as the number of new cases in *V* adjacent (connected by an edge) to any known case in *V*^c^, where R≤|V|. To resolve the event that a new vertex in *V* is adjacent to vertices in more than one cluster, we reduced the subset of edges between *V* and *V*^c^, maintaining only the edges with minimum weight per vertex in *V*. If more than one edge to a given vertex u∈V had exactly the same minimum weight, then we selected one edge at random.

We formulated two predictive models to generate estimates of growth for the *i*th cluster *C_i_*, which we denote respectively as the null model and the weighted model. The null model requires less information by postulating that each cluster is expected to grow in proportion to its current size, prior to the addition of new cases, as a fraction of the entire population of known cases. For example, a cluster that comprises half of all known cases is predicted to accumulate half of new cases that are adjacent to any cluster. Expressed as a Poisson regression model, the expected growth of *C_i_* given total cluster growth *R* is given by:

(1) $$E\left(R_{0}(C_{i})\right)=\text{exp}\;\left(\frac{\left|C_{i}\right|}{\left|V^{c}\right|}R\right).$$

where we use boldface **E** to denote the expectation (and distinguish it from our edge set notation *E*), and *R*_0_ is given the subscript 0 to indicate it is the predicted growth under the null model. Thus the null model does not use any individual-level attributes to predict cluster growth—it is a naive model that assumes that the allocation of cluster-adjacent new cases in *R* is not influenced by any characteristics of those clusters other than the ‘space’ they occupy. In contrast, the weighted model assigns individual-level weights *w*(*v*) to every vertex in $v\in C_{i}$. Expressed as a Poisson regression model, the expected growth of cluster *C_i_* under the weighted model is written:

(2) $$E(R(C_{i}))=\text{exp}\;\left(\alpha +\beta \sum_{v\in C_{i}}w(v)\right)$$

where *α* and *β* are parameters to be estimated by regression. Note that Equation (2) reduces to (1) when *w*(*v*) = 1 for all $v\in C_{i}$, *α*  =  0 and $\beta =R/|V^{c}|$. There are two advantages to using individual-level weights in Equation (2) rather than cluster-level weights: first, individual-level weights are independent of clustering thresholds, so it is sufficient to calculate weights one time only; second, individual-level weights confer a greater degree of precision for measuring effects on edge densities that would become averaged out in clusters.

For our demonstration, we weighted individual cases by their recency of sample collection or diagnosis, measured as $\Delta t=t_{\max}-t(v)$. The predictive weight *w* of a known case of a given age Δt relative to *t*_max_ was based on the expected rate of adjacency (edge density) between sets of known cases separated by the same time lag. Thus, we needed to calculate the edge densities for all bipartite graphs $K_{ij}=(V_{i}^{c},V_{j}^{c},E_{ij})$ where $V_{i}^{c}=\{v\in V^{c}:t(v)=i\}$ and j−i=Δt. For compatibility with our definition of cluster growth, we removed bipartite edges from *E_ij_* so that the maximum degree size for any vertex $v\in V_{j}^{c}$ was 1, where the remaining edge minimized the edge weight *w*(*u*, *v*) for all *u* given *v*. We use $E_{ij}^{1}$ to denote this reduced set of bipartite edges. This had the effect of reducing the maximum possible number of bipartite edges in *K_ij_* from $\left|V_{i}||V_{j}\right|$ to $\left|V_{j}\right|$. We refer to the set of all bipartite graphs for a given time lag as $K(\Delta t)=\{K_{ij}:j>i,j-i=\Delta t\}$. Thus, the expected edge density *ρ* given Δt is:

(3) $$E(\rho |\Delta t)=\frac{\sum_{K(\Delta t)}|E_{ij}^{1}|/|V_{j}^{c}|}{(t_{\max}-1)-t_{\min}-\Delta t}$$

where the denominator adjusts for the number of bipartite graphs with time lag Δt. For this model, we assume that the edges in $E_{ij}^{1}$ are independent and identically distributed binary outcomes. Furthermore, we expect the probability of this outcome to decay with increasing Δt. Hence we used logistic regression to estimate *ρ* as a function of Δt:

(4) $$\text{log}\;\left(\frac{\hat{\rho }}{1-\hat{\rho }}\right)=\alpha \prime +\beta \prime \Delta t$$

where α′ and β′ are parameters to be estimated by regression. Using logistic regression enabled us to measure the effect of case recency at the level of individuals. The simplest use of this information would be to set the weight of a known case to its predicted edge density given its time lag Δt relative to the most recent year; i.e. $w(v)=\hat{\rho }(\Delta t)$. To summarize, we identified edges that minimized the genetic distance between known cases separated by a given time lag (Equation 3) and fit a logistic model to these edge densities as a function of time lag (Equation 4) so we can predict the adjacency of new cases to a cluster of known cases given their net recency.

The weighted model can be extended to employ a linear combination of additional individual-level attributes (e.g. plasma viral load) and/or graph attributes that are parameterized from bipartite subgraphs on *V*^c^. For example, we added an additional measure, deg(v), that represents the average degree of known cases from the same time point as *v*. A high mean degree in the graph for a given threshold $d_{\max}$ can reveal specific time points with an unusually dense concentration of cases in genetic space, which may be caused by a period of increased sampling effort or a past outbreak. Without making some adjustment, this period can have a disproportionate influence on the association of edge density on Δt as estimated by Equation (4). Thus we also evaluated a weighted model substituting the following weighting formula into Equation (2):

(5) $$w(v)=\beta_{0}\hat{\rho }(t_{\max}-t(v))+\beta_{1}\deg(v)$$

where the coefficient *β* has been brought into the summation over individual known cases. In this model, deg(v) is a graph-level attribute that controls for the confounding effect of variation in degree size among years of diagnosis or collection. We have released R scripts and examples for calculating generalized Akaike information criterion (GAIC) profiles under a permissive free software license at https://github.com/PoonLab/MountainPlot (last accessed 20 January 2020).

### 2.4 Evaluating cluster thresholds

For each data set, we segregated all HIV-1 sequences that were sampled in the most recent year as new cases comprising the set *V*. Next, we extracted the observed cluster growth outcomes $R(C_{i})$ and individual case weights *w*(*v*) at fifty-one different cluster-defining distance thresholds, ranging from $d_{\max}=0$ to $d_{\max}=0.04$ in steps of $8\times 10^{-4}$. To clarify, we used the same threshold $d_{\max}$ to evaluate cluster growth (the occurrence of new cases in clusters) as was used to generate the clusters of known cases. We fit the null and weighted models described by Equations (1) and (2) to the resulting distributions of cluster growth. The Akaike information criterion (AIC), which penalizes likelihood for the number of model parameters, was recorded for each regression model (Akaike 1998). The ‘generalized AIC’ (GAIC) is simply the difference in AIC between models, and has been proposed as a key quantity for resolving the MAUP (Nakaya 2000). Cut-offs with a negative GAIC indicate that the weighted model explains the data more effectively than the null model, and the magnitude of GAIC quantifies that difference in effectiveness. We define the optimal distance threshold as the value $d_{\max}$ associated with the lowest (most negative) GAIC. The GAIC was evaluated for the weighted models using either dates of sample collection or diagnosis to compute Δt for the Middle Tennessee data set for which both dates were available. Since some dates were missing data, we normalized the number of new cases to |V|=125 for both analyses to ensure that growth rates were comparable. After this step, the total number of cases was reduced to *n *=* *2,015 and *n *=* *2,588 for the diagnostic and sample collection analyses, respectively. We note that the resulting weighted models are not being compared directly; instead, they are compared to the null models for their respective data sets.

We repeated the cluster threshold evaluation on progressively censored subsets of the Seattle and Tennessee data to evaluate the consistency of the GAIC-optimized thresholds over time. This was accomplished by removing cases from the most recent year to a maximum of four years, and obtaining the GAIC measurements for all values of $d_{\max}$ for the remaining data at each step. Because of the limited size and temporal range of the northern Alberta data set, we did not use it for this sensitivity analysis. We also re-ran the experiment on random subsets of cases from the complete Seattle and Tennessee data sets, creating a total of ninety subsets; thirty subsets at three different proportions of the original total, 80 per cent, 60 per cent, and 40 per cet. For each resampling proportion, we obtained the kernel density for the optimal cut-off location over the thirty replicate samples and the GAIC measurements obtained by a smoothed function from all ninety resampled GAIC runs.

## 3. Results

### 3.1 Study populations

A total of *n *=* *5,010 HIV-1 sequences and sample collection dates were obtained from published studies in Seattle (*n *=* *1,591; Wolf *et al.* 2017), northern Alberta (*n *=* *803; Vrancken *et al.* 2017), and Middle Tennessee (*n *=* *2,616; Dennis *et al.* 2018), respectively. In addition, dates of HIV-1 diagnosis were available for a total of 2,527 cases in the Tennessee data set. The distributions of sample collection dates are summarized in Fig. 1 and the direct comparison between diagnostic and collection year distributions for the Tennessee data set can be found in Supplementary Fig. S1. The lowest mean sampling rate was obtained in northern Alberta, with 114.7 cases per year, compared to 122.4 and 174.4 cases/year for Seattle and Tennessee, respectively. Cases from the final years of sampling were omitted to adjust for studies being terminated before the end of the calendar year. For instance, there were only thirty-five cases sampled in 2013 in the Seattle data set, where sample collection was ended on March 2013 (Wolf *et al.* 2017)—since the cases in the final year were reserved to evaluate the predictive models, an artificially low sample size would have a disproportionate influence on model validation. Hence, we proceeded with 110 cases collected in 2012, 110 cases from 2013, and 153 cases from 2015 for Seattle, northern Alberta, and Tennessee, respectively. We refer to the cases sampled in these final years as ‘new cases’, and those sampled in the preceding years as ‘known cases’.

For each data set, we calculated the TN93 (Tamura and Nei 1993) genetic distance for every pair of sequences. This distance, which adjusts for differences in the mean rates among nucleotide transversions and the two types of transitions, is the basis for clustering in the HIV-TRACE programme (Pond *et al.* 2018) that is employed by the US-CDC for public health surveillance (Oster *et al.* 2018). Although the northern Alberta data set comprised a smaller number of sequences, the lower tail of its TN93 distribution contained relatively higher numbers of pairwise distances than the other two data sets (Fig. 1). For instance, the TN93 distance at the 1 per cent quantile was 0.013 in northern Alberta, while the same quantile was roughly twice this distance for Seattle (0.026) and Tennessee (0.023). Overall, these distributions were significantly different (Kruskal–Wallis test, $P<10^{-15}$).

### 3.2 Adjacency of cases decays with time lag

We generated a sequence of graphs at varying TN93 distance thresholds for each data set, where each vertex represents a known case (sampled or diagnosed prior to the final year) and an edge indicates that the corresponding pairwise distance is below the threshold—in graph theory, the cases are said to be ‘adjacent’. Thus, each distance threshold defines a different partition of known cases into clusters, where a cluster may consist of only a single known case. Our objective is to determine which threshold results in the most information-rich partition of known cases for predicting where new cases will arise. As we will demonstrate below, there is no information value in either extreme of a single giant cluster or the complete atomization of cases into singular clusters. To quantify the information loss associated with different partitions, we compared two predictive models. First, we fit a null model that assumes the probability that a new case appears in a cluster (i.e. cluster growth) is only influenced by the number of known cases in the cluster, i.e. the cluster size. This is equivalent to assuming that every known case is equally likely to be adjacent (connected by an edge) to the new case. Second, we fit a weighted model where the probability of cluster growth is predicted by some linear combination of individual-level attributes among the known cases in the cluster.

**Figure 2.**
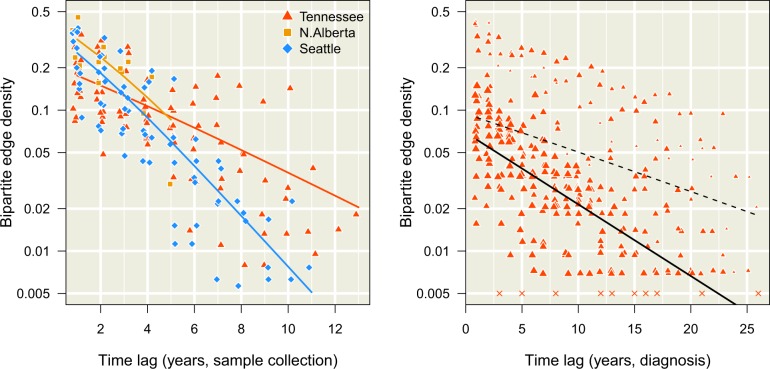
Decay in bipartite edge densities with increasing time lags. Each point represents a bipartite graph at a given time lag (difference in years, *x*-axis), coloured with respect to the data set (see inset legend). We added random noise along the horizontal axis to separate overlapping points. The log-transformed *y*-axis represents the frequency of edges between cases in different time points below the threshold *d*_max_ = 0.04 (Equation 3). (left) Decay of edge densities with respect to dates of sample collection. Each trend line summarizes the binomial regression model (Equation 4) for each data set. (right) Decay of edge densities with respect to dates of HIV diagnosis in Middle Tennessee. Each point was scaled in proportion to the mean degree size among known cases in the earlier time point. Bipartite graphs without any edges are represented by × marks at the arbitrary value of 0.005, since zero cannot be displayed on a log-transformed axis. The dashed line indicates the fit of the binomial regression to these data, while the solid line indicates the fit of an extended model (Equation 5) that controls for variation in the mean degree size.

For example, we hypothesize that the probability that a new case is adjacent to a known case declines with an increasing time lag between their respective sample collection dates. To investigate this effect, we plotted the observed densities of edges at a threshold $d_{\max}=0.04$ between sets of known cases sampled in different years (Fig. 2). These plots confirm that edge densities decline significantly with increasing time lag (Δt), which we measured by fitting binomial regression models (Equation 4). Specifically, the estimated effect of Δt on the log-odds of a bipartite edge was –0.42 (95% CI $=-0.45,-0.39)\;{\text{year}}^{-1}$ for the Seattle data and $-0.40\;(-0.48,-0.32)\;{\text{year}}^{-1}$ for northern Alberta. The coefficient of determination for the respective models was $R^{2}=0.70$ and 0.58. For the Tennessee data set, the effect of time lag was lower than the other data sets (−0.19 (−0.21,−0.18) year^–^^1^; $R^{2}=0.41$). In general, lowering $d_{\max}$ reduced the observed bipartite edge densities as fewer edge weights passed the threshold. Nevertheless, the negative associations between Δt and the log-odds of bipartite edges were robust to varying $d_{\max}$ (Supplementary Fig. S2). These results supported the use of ‘case recency’ (the time lag between sample collection dates) as an individual-level predictor of a new case joining a cluster of known cases.

Since dates of HIV-1 diagnosis were also available for the Tennessee data set, we applied the same binomial regression to known cases separated by their years of HIV diagnosis rather than by sample collection (Fig. 2, right). We noted that the decline in adjacency rates with time lag was distorted by unusually high edge densities involving cases diagnosed in 1982, 1984 and 1986, which resulted in a much smaller but significant effect of Δt (−0.067 (95%C.I.=-0.074,-0.060) year^–^^1^; $R^{2}=0.016$). This motivated the use of an extended binomial regression model (Equation 5) to control for variation in degree size among years of diagnosis for known cases. We found that controlling for variation in degree sizes among years substantially improved the fit of the regression model to the time lags in diagnosis dates ($R^{2}=0.37,\;\Delta \mathrm{AIC}=-351.4$). Moreover, this extended model conferred significantly improved fits to sample collection dates for all three data sets (ΔAIC=−7.5, –12.8 and –171.6 for Seattle, northern Alberta and Middle Tennessee, respectively). Therefore, we used the extended binomial regression model for our subsequent analyses to predict the distribution of new cases among clusters.

### 3.3 Trade-off between case coverage and cluster information

**Figure 3.**
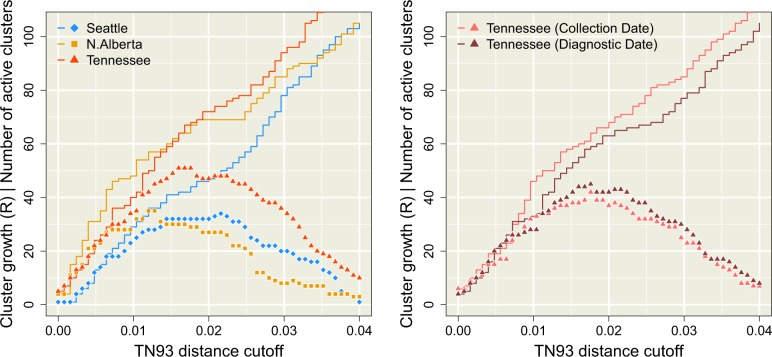
Distribution of new cases among clusters as a function of the Tamura-Nei (TN93) distance clustering threshold ($d_{\max}$, *x*-axis). The solid lines represent the total number (*R*) of new cases adjacent to clusters of known cases. The points correspond to the number of clusters of known cases with edges to new cases, which we refer to as ‘active’ clusters. (left) This plot summarizes the trends obtained when cases are stratified by year of sample collection. Trends in *R* and numbers of active clusters are coloured with respect to data set (see inset legend). (right) This plot contrasts the trends obtained from the Tennessee data set when cases were stratified by year of sample collection (lighter red) versus the year of diagnosis (darker red). Note that the collection date trend is not identical to the trend in the left plot because we downsampled cases to match the availability of diagnosis dates.

Figure 3 illustrates the effect of relaxing the threshold $d_{\max}$ on the number of new cases that are adjacent to one or more known cases, which we denote by *R*. A new case that is not adjacent to any known case cannot be anticipated by any clustering method. Although these results do not inform us about our ability to forecast cluster growth, i.e. which cluster of known cases is more likely to accumulate new cases, they do characterize the effect size of the TN93 distance cut-off for perceived cluster growth. When *R* approaches the total number of new cases (denoted as |V|, where *V* is the set of vertices in the final year) we say that the clusters have a high *case coverage*. As expected, decreasing $d_{\max}$ reduced *R* as a progressively greater number of edges were excluded, which would limit the utility of clustering for public health surveillance. The accumulation of *R* with increasing $d_{\max}$ was slightly slower for Seattle than the other data sets (Fig. 3, left), which was not anticipated by our comparisons of the overall distributions of TN93 distances among data sets (Fig. 1, bottom). In addition, Fig. 3 (left) summarizes how the number of clusters with at least one edge to a new case (the number of active clusters) initially tracks the accumulation in *R* with increasing $d_{\max}$. Thus *R* sets an upper limit to the number of active clusters; these numbers can only be equal if each new case is uniquely adjacent to its own cluster, as we observed for the Seattle data set for cut-offs below $w_{\max}=0.0072$ or the Tennessee data at cut-offs below $w_{\max}=0.008$. As $d_{\max}$ continues to increase, the number of active clusters peaks and begins to decline towards 1. This outcome reflects the gradual accretion of cases into a single giant cluster. Substituting year of diagnosis for sample collection dates in the Tennessee data resulted in a slight reduction in *R* and a modest increase in the number of active clusters, which we attribute to a more uniform distribution of new cases across clusters.

### 3.4 Obtaining GAIC

The results in the preceding section imply that there exists an intermediate value of $d_{\max}$ that optimizes the trade-off between case coverage and the number of active clusters, where both quantities have a significant impact on the information content of clusters for public health. We propose that the best criterion for optimizing a clustering method is our ability to predict where the next cases will occur among the resulting clusters. Specifically, we adapted the GAIC (Nakaya 2000) to select the optimal threshold. Our implementation of the GAIC is a comparison between two Poisson regression models, where the count outcomes are the number of new cases adjacent to each cluster. In the null model, we assume that the rate parameter is proportional to cluster size as a fraction of all known cases (Equation 1), assuming no variation among individual known cases. In the weighted model, the rate parameter is the total weight of known cases in the *i*th cluster, where each weight can be calculated from a linear combination of individual- or group-level attributes. For our analysis, we weighted cases by their predicted edge densities from the extended binomial model (Equation 5).

**Figure 4.**
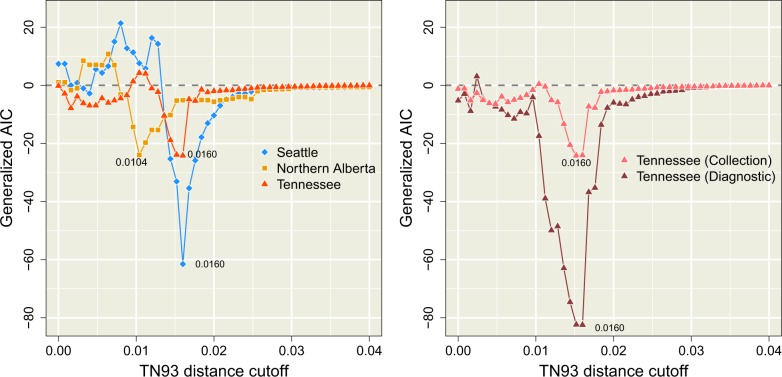
Profiles of GAIC relative to the distance cut-off $d_{\max}$. This is calculated as the difference in AIC between the weighted and null Poisson models. A negative GAIC value indicates that the weighted model is more effective in predicting the distribution of new cases among the clusters defined at that $d_{\max}$. (left) Profiles for the three complete data sets using collection dates. The global GAIC minima for each data set are labelled with the corresponding TN93 distance cut-off values. We note that GAIC values become more erratic with decreasing $d_{\max}$ as the distribution of new cases among smaller clusters becomes increasingly sparse and stochastic. (right) Profiles for the Tennessee data sets using sample collection or diagnosis dates, excluding cases with missing diagnostic dates for direct comparison.

Figure 4 (left) summarizes the distributions of GAIC for varying $d_{\max}$ for each data set. We observed that GAIC tended to be near zero for relaxed thresholds ($d_{\max}\geq 0.03$), which indicated that the ability of the weighted model to forecast new cases was indistinguishable from the null model. At these high thresholds, the majority of known cases tended to become grouped into a single large cluster, thereby homogenizing any individual-level variation that could be used by the weighted model to predict the distribution of new cases. The minimum (most negative) GAIC was obtained $d_{\max}=0.0104$ for northern Alberta and $d_{\max}=0.0160$ for both Seattle and Tennessee. These minima identified the optimal thresholds that maximized the difference in information loss between the weighted and null models. As we continued to decrease $d_{\max}$ past these optima, the GAIC trends returned to the zero line and even increase sporadically into positive values where the weighted model was *worse* than the null model. At these low values of $d_{\max}$, the disintegration of clusters into large number of singletons disrupts the covariation between individual attributes and the distribution of new cases. Furthermore, lowering $d_{\max}$ also leads to a reduction of case coverage as shown in Fig. 3. At the respective optimal thresholds, less than half of new cases were adjacent to clusters of known cases (38.2% for Seattle, 49.1% for Northern Alberta, and 41.8% for Tennessee).

The GAIC also provides a framework for variable selection. For instance, the GAIC obtained for the Tennessee data when cases were stratified by year of diagnosis was substantially lower than the values obtained with sample collection dates over wide range of $d_{\max}$ (Fig. 4, right). The optimal threshold identified by the minimum GAIC coincided for both sets of dates ($d_{\max}$); however, the GAIC for diagnosis dates was substantially more negative (ΔGAIC=−58.3), indicating a more effective use of cluster information. For larger values of $d_{\max}>0.02$, the stratification of cases by either set of dates was irrelevant as they collapsed into a single giant cluster, such that both GAIC trends converged to zero. For smaller values of $d_{\max}<0.01$, both trends became more erratic with the reduced adjacency of new cases and increasingly stochastic composition of smaller clusters.

**Figure 5.**
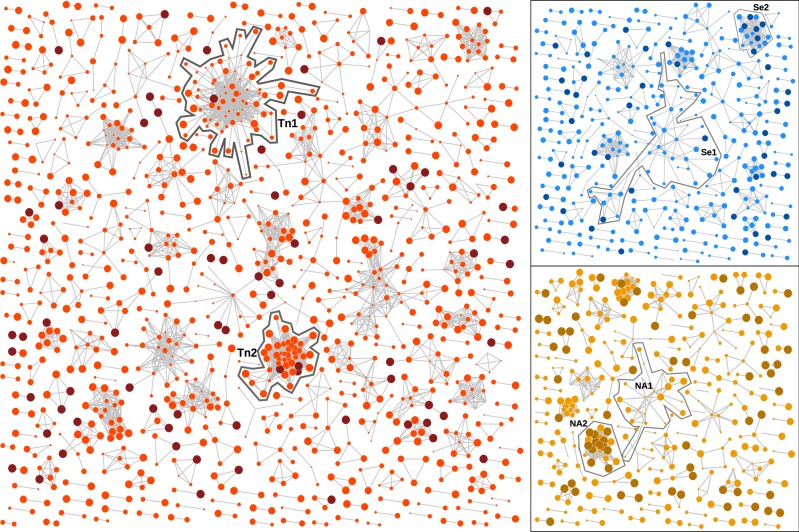
Network visualizations of the graphs from the Middle Tennessee data (red), Seattle (blue), and northern Alberta (orange) data sets obtained at the respective GAIC-optimized distance thresholds, rendered using an implementation of the Kamada and Kawai (1989) algorithm. Vertices that corresponded to new cases were coloured in a darker shade, and the width of each vertex was scaled to the sample collection year with more recent cases drawn at a larger size. The vertices with zero degrees (singletons) were not drawn for clarity. Clusters with the largest number of new cases and/or the largest number of cases overall are highlighted for discussion in the text.

The graphs for these optimal cut-offs are summarized in Fig. 5. In the Seattle graph, the largest cluster (Se1) comprising thirty-four known cases was adjacent to two new cases. The sample collection years associated with this cluster range from 2000 to 2012 with a mean of 2006.8 (interquartile range, IQR = 2006–2009). In contrast, the second largest cluster (Se2) comprised only ten known cases that were sampled more recently with a mean of 2009.6 (2008–2011), and accumulated six new cases. Similarly, the largest cluster in the northern Alberta graph (NA1) comprised twenty-two cases of which none were new, with a mean sample collection year of 2009.2 years (2008–2011). This contrasts a smaller cluster of ten known cases (NA2), of which five were collected in 2012 (mean 2010.5, IQR = 2009–2012), that gained twelve new cases in 2013. Finally, we observed a large cluster (Tn1) of seventy-two known cases in the Tennessee data set with only one new case and a mean sample collection year of 2007.6 (IQR = 2005–2010). In contrast, the cluster labelled Tn2 comprised thirty-nine known cases with a mean collection year of 2012 (IQR 2010, 2013) and three new cases. These simple examples illustrate the effect of optimizing the clustering threshold on the covariation of new case counts and the recency of known cases among clusters. As predicted by our model analysis, the effect of variation in case recency among clusters on the distribution of new cases is even clearer for the graph depicting clusters of cases stratified by year of diagnosis rather than sample collection (Supplementary Fig. S3).

### 3.5 Robustness of GAIC optimization

The difference in optimal clustering thresholds between northern Alberta and the other sites implies that a globally optimal threshold does not exist. However, variation in thresholds may also be a stochastic outcome due to incomplete sampling. To measure the effect of sampling variation, we repeated our GAIC analysis on random sub-samples of the Seattle and Tennessee data sets to 40 per cent, 60 per cent, and 80 per cent of the data (thirty replicates each). The comparably low number of cases per year in the northern Alberta data set precluded sub-sampling. The results for the Seattle data set are summarized in Supplementary Fig. S4. As expected, we observed shallower GAIC trends and more variable GAIC-optimized thresholds with decreasing sample size. However, the optimal thresholds remained clustered around the original value $d_{\max}=0.016$, with only four (13%) replicates with optima below $d_{\max}<0.015$ at 40 per cent sub-sampling (*n *=* *636, comparable to the northern Alberta sample size of *n *=* *803). We obtained similar results on random sub-samples of the Tennessee data set, except that the optima were more robust for the data stratified by diagnosis dates.

**Figure 6.**
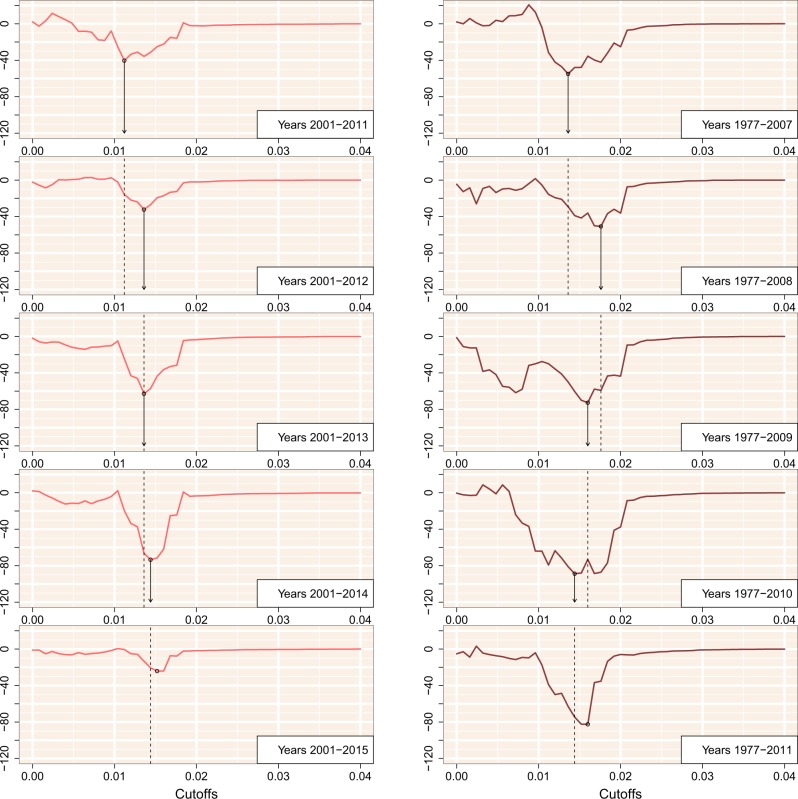
GAIC plotted against the cut-off threshold for five progressively right-censored subgraphs with respect to sample collection (left) and diagnosis (right) for the Middle Tennessee data set. The minimum GAIC for each plot is indicated by a circular mark. Arrows and dashed lines indicate where the GAIC-optimized threshold from the previous result would carry over to the subsequent year.

Finally, to assess the stability of GAIC-optimized thresholds over time, we generated additional sub-samples by progressively right censoring the Seattle and Tennessee data sets. If the information content of pairwise TN93 clusters is stable over time, then we should expect the optimized threshold from fitting models to ‘new cases’ in a given year should be similar to the optimized threshold with an additional year of case data. Figure 6 summarizes our results for the Tennessee data set where the four most recent years were progressively censored by dates of sample collection (left) and diagnosis (right), respectively. Results for the complete Tennessee data set (including cases with missing diagnosis dates) and the Seattle data set are provided in the Supplementary Fig. S7. In general, we observed that the optimal threshold identified by the minimum GAIC was relatively stable over time. Although the exact thresholds varied slightly, the optimal threshold from the previous year tended to confer a GAIC similar to the threshold estimated from that year. In our analysis of the Seattle data set, however, the optimal distance thresholds fluctuated between two local minima around $d_{\max}=0.005$ and 0.015. For instance, in the subset where cases were limited to 2011 (censoring cases sampled in 2012) we observed three local minima in the GAIC ($d_{\max}=0.0024$, 0.0072, and 0.0144), including one that was close to the global minimum of the previous year. These results suggest that the larger database of cases sampled in Middle Tennessee confer increased robustness to sampling variation, although we cannot rule out site-specific effects.

## 4. Discussion

Our results demonstrate that an apparently small difference in pairwise genetic distances—for instance, between 0.5 per cent and 1.5 per cent—can make the difference between accurate forecasting of new cases among clusters and becoming misled by stochastic noise. Specifically, both cut-offs cited above are routinely used as customary settings in pairwise genetic distance clustering studies of the same HIV-1 subtype in the same country (Oster *et al.* 2015; Wolf *et al.* 2017; National Center for HIV/AIDS, Viral Hepatitis, STD, and TB Prevention, 2018). To investigate the sensitivity of clustering thresholds, we have examined three data sets of anonymized HIV-1 subtype B *pol* sequences that were collected in different regions of North America within similar time frames. All GAIC-optimized cut-offs are located in the left tails of the respective empirical distributions of pairwise distances, with no clear demarcation that might motivate the selection *a priori* of one cut-off over another (Fig. 1). However, our information-based criterion reveals a stark difference between these cut-offs when we evaluate the ability of genetic clusters to ‘forecast’ the occurrence of new cases (Fig. 4). This discordance is a result of a trade-off between the coverage and predictive value of spatial information that is encapsulated by the MAUP (Nakaya 2000). As we relax the clustering threshold, instances of cluster growth become more frequent such that aggregate effects (viz. case recency and mean degree) can be distinguished against the background of stochastic effects. However, the variation in growth rates among clusters eventually becomes homogenized as they are collapsed into a single giant cluster at increasingly high thresholds. These two driving forces can be differentiated in their approach to an optimum, with poor case coverage from strict thresholds resulting in erratic changes in forecasting information, and higher case coverage with relaxed thresholds resulting in a smoother gain of information. If the database is small relative to the population or the number of new cases per year is low, the global minimum in the GAIC profile may become obscured by sampling variation (Supplementary Fig. S4), especially when the true optimal threshold is low. Resampling experiments (Supplementary Figs S4–S6) can help ameliorate the effects of sampling variation and identify situations where sample size may be a concern.

Unlike most instances of the MAUP that arise in spatial epidemiology (Nakaya 2000; Swift, Liu, and Uber 2008; Parenteau and Sawada 2011; Haddawy *et al.* 2018), our outcome variable (the number of new cases per genetic cluster) is directly dependent on the same parameters that reshape the partition of the spatial distribution of covarates into units. This dual dependency results in an asymptotically increasing model likelihood with increasing distance thresholds, plateauing at the point where all known cases were assigned to the same giant cluster, such that any new cases are effectively guaranteed to be adjacent to this cluster. We addressed this unique problem by formulating a null model where the predicted growth of a cluster was directly proportional to its relative size in the number of known cases. Hence, this null model provided a useful baseline that controlled for the proportionate effect of the largest cluster with increasing cut-offs, thereby enabling us to focus on the predictive value of variation in covariates among clusters.

We selected a relatively simple clustering method in widespread use (pairwise TN93 distance clustering by components; Aldous *et al.* 2012) to demonstrate our new framework for evaluating clusters, which is based on the GAIC proposed by Nakaya (2000). Pairwise clustering has been widely adopted in health jurisdictions around the world, including the US-CDC (Oster *et al.* 2018), in part due to the growing popularity of the HIV-TRACE software package that employs TN93 (Pond *et al.* 2018). However, we hypothesize that it should be feasible to use this framework to evaluate potentially any clustering method that defines a partition on the database of known cases. For example, if we require some minimum bootstrap support value to define clusters as subtrees extracted from the total phylogenetic tree (Ragonnet-Cronin *et al.* 2013), then this bootstrap support threshold can represent a second dimension (in addition to a branch length threshold) to locate the minimal GAIC in combination with a distance threshold. We have also demonstrated that the GAIC can be repurposed for model selection where different linear combinations of predictor variables, such as the mean degree size in a given year of diagnosis (Fig. 4), are evaluated within the Poisson regression model. Billock *et al.* (2019) recently employed a similar model selection approach for pairwise TN93 clusters, although their analysis pre-specified a fixed clustering threshold of 1.5 per cent. Thus, if sufficient metadata are available then one can use the GAIC to select more accurate predictive models of cluster growth while adjusting the clustering criteria, so the models can be evaluated at their best performance.

Collection dates in units of years are most frequently available as sample metadata in association with published HIV-1 genetic sequences. We had a strong *a priori* expectation for an association between new case adjacency and known case recency that we subsequently confirmed from these data (Fig. 2). On the other hand, we recognize that samples may be collected well after the start of a new infection, due to the long asymptomatic period of HIV-1 infection and social barriers to HIV testing (Mahajan *et al.* 2008). Even when using diagnostic dates, there is potential conflation of recent transmission with late diagnosis. Although estimated dates of infection (e.g. the midpoint between the last HIV seronegative and first seropositive visit dates) will tend to be closer to the actual date of infection, the necessary information for accurate estimates are not routinely available in a public health context. We furthermore recognize that more precise dates of sample collection would likely confer greater prediction accuracy. Indeed, the granularity of time in the context of genetic cluster analysis represents an extension of MAUP that is known as the modifiable temporal unit problem (Cheng and Adepeju 2014). While reducing the length of time intervals may produce more timely predictions, e.g. new cases in the next three months instead of the next year, the accuracy of prediction will erode with progressively shorter intervals. Finally, we propose that an informative assessment on the potential value of genetic clustering for public health would be to compare the GAIC of the genetic clustering method against the value obtained from the prioritization of groups by public health experts. However, the confidential information comprising the latter case is not likely to be found in the public domain.

An important caveat to our approach is that the expected probability of an edge between specific known and new cases is very small. Consequently, our method requires a substantial number of new cases to parameterize models of the variation in edge densities among clusters and, ultimately, to discriminate between the null and weighted models. (Note that the number of cases sampled in a given year does not correspond to the annual incidence.) Given the results summarized in Fig. 6, if those requirements are not met, the minimal GAIC that results from the theoretical MAUP trade-off may be masked by the initial noise of a graph with low case coverage and the merging of clusters with increasing thresholds. The results that we obtained with the smallest data set (Northern Alberta) and our sub-sampling experiments (Supplementary Figs S4–S6) imply that averaging about 50–100 sampled cases per year over a 6-year period should be adequate for the relatively simple models evaluated here. In addition, our comparison of sample collection versus diagnosis dates (Supplementary Figs S5 and S6) implies that the minimum sample size requirement should diminish with the addition of covariates into the weighted model that have strong associations with the distribution of new cases.

Genetic clustering is used increasingly for near real-time monitoring of clinical populations for the purpose of guiding public health activities (Poon *et al.* 2016; Rose *et al.* 2017; Oster *et al.* 2018; Billock *et al.* 2019). Our method is not specific to HIV-1, although the proliferation of clustering methods in HIV molecular epidemiology—driven by the abundance of genetic sequence data and the relatively rapid rate of evolution and low transmission rate of the virus—does make this approach particularly applicable to this virus. Similar pairwise distance clustering methods, for instance, have been used for *Mycobacterium tuberculosis* (Walker *et al.* 2013) and hepatitis C virus (Jacka *et al.* 2014) to infer epidemiological characteristics from molecular sequence variation. In these cases, it may be necessary to rescale the step size/range of clustering thresholds to the expected distribution of genetic distances in order to locate the minimum GAIC. Furthermore, variation in population density and overall case prevalence among regions, as well as the proportion of diagnosed cases, will influence the GAIC. For instance, the visible difference in minimum GAIC we observed between data sets from Northern Alberta and the two locations in the USA was likely driven by differences in population density and sampling fraction. Applications of clustering for HIV-1 prevention have tended to be developed in the context of low-level epidemics, e.g. North America and western Europe, making it challenging to translate these methods to generalized epidemics in other countries without a robust statistical framework for calibrating methods. No matter what pathogen or location is the focus of investigation, it is imperative that we become more critical of clustering methods (Volz *et al.* 2012; Poon 2016). Improperly calibrated clustering methods may otherwise prioritize false positives, diverting limited public health resources away from subpopulations where the immediate need for prevention and treatment services was greatest.

## Supplementary Material

veaa011_Supplementary_Data

## Data Availability

Data available at GenBank accession numbers KY034691–KY037792, KU190031–KU190839 ,and MH352627–MH355541.
