# Supplementary figures and images for: Public health in genetic spaces: a statistical framework to optimize cluster-based outbreak detection
Source: Virus Evol. 2020 Mar 13;6(1):veaa011. doi: 10.1093/ve/veaa011 (PMC7069216; doi:10.1093/ve/veaa011)

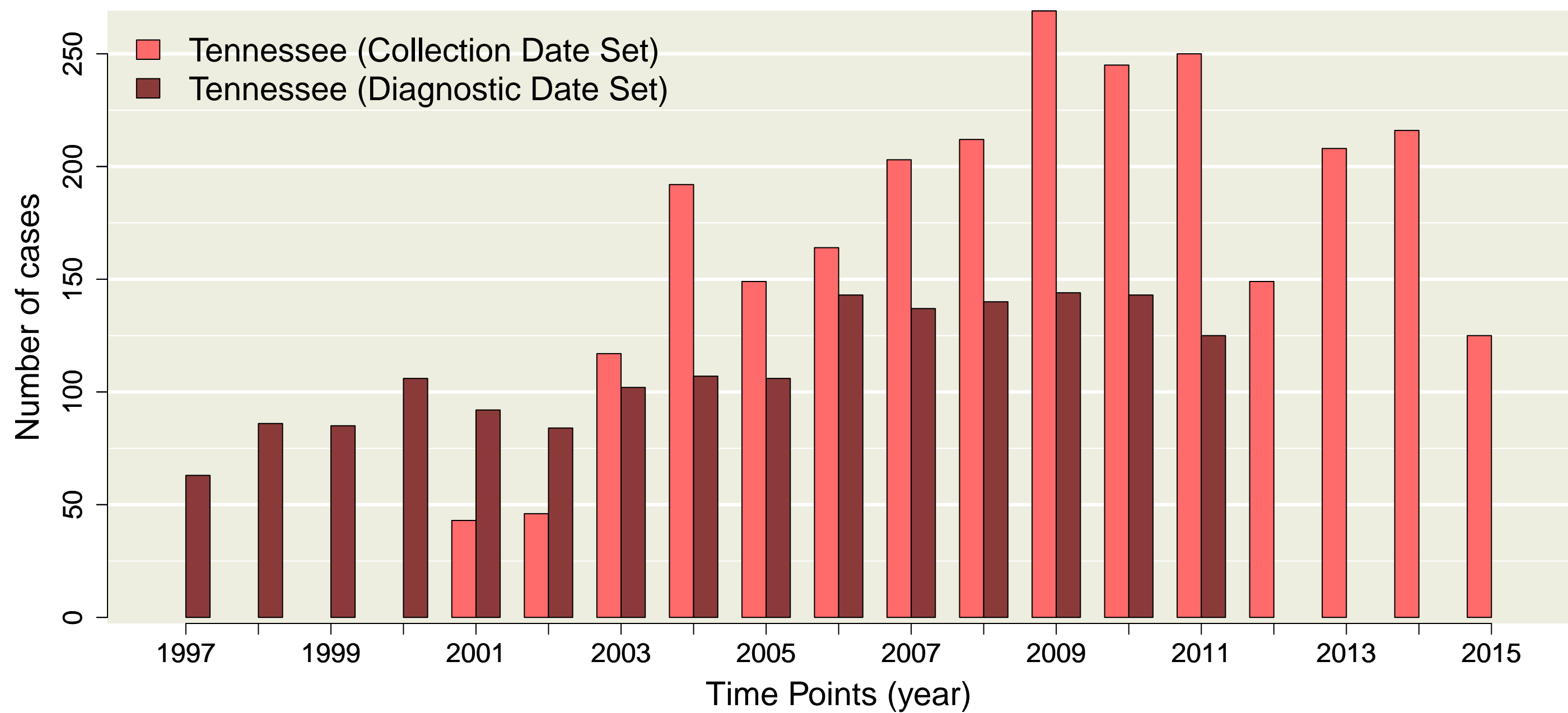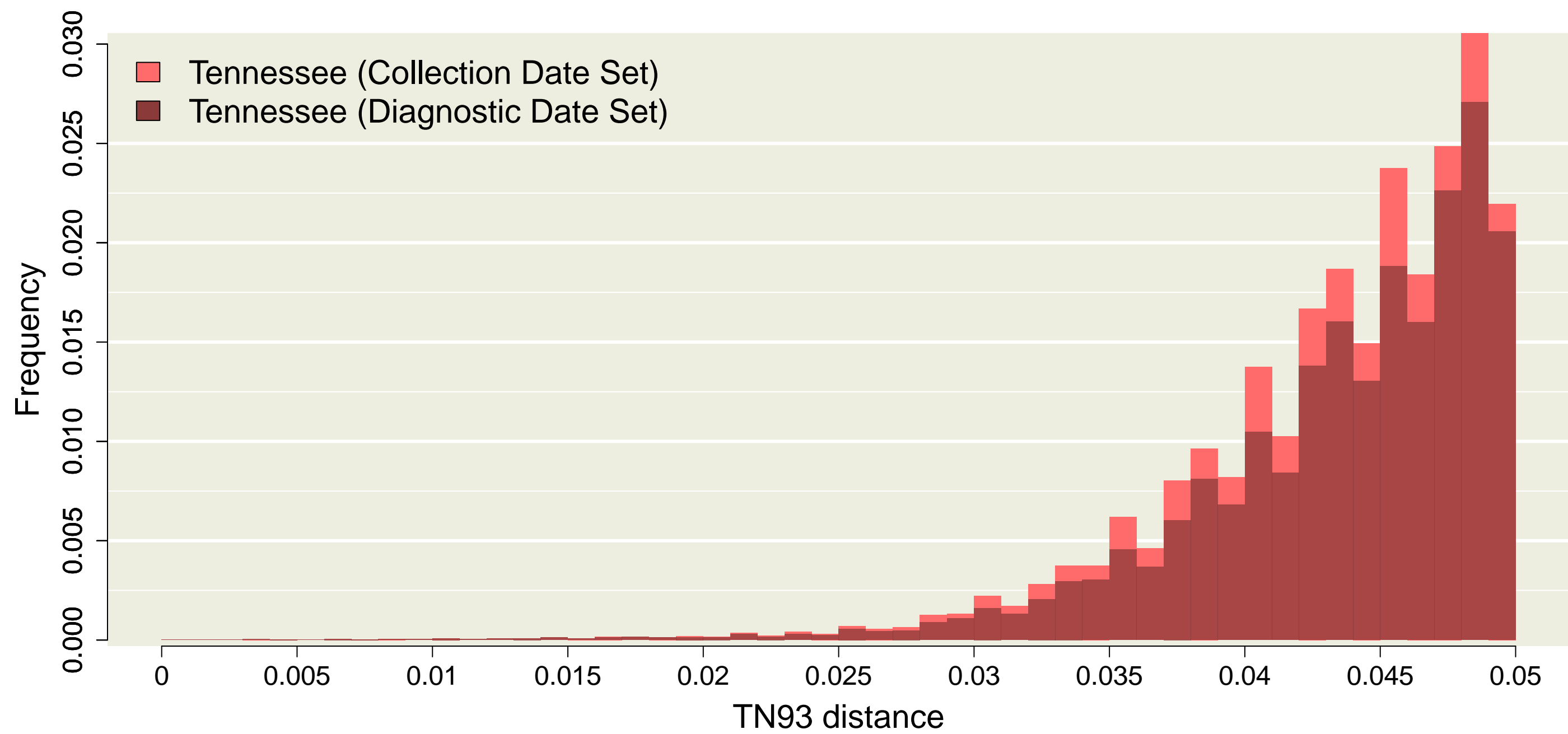

Supplement: veaa011_Supplementary_Data [file veaa011_supplementary_data.pdf]
